# Supplementary material for: The protein structurome of Orthornavirae and its dark matter
Source: mBio. 2024 Dec 23;16(2):e03200-24. doi: 10.1128/mbio.03200-24 (PMC11796362; doi:10.1128/mbio.03200-24)

Supplementary Figures

Fig. S1: Workflow schematic

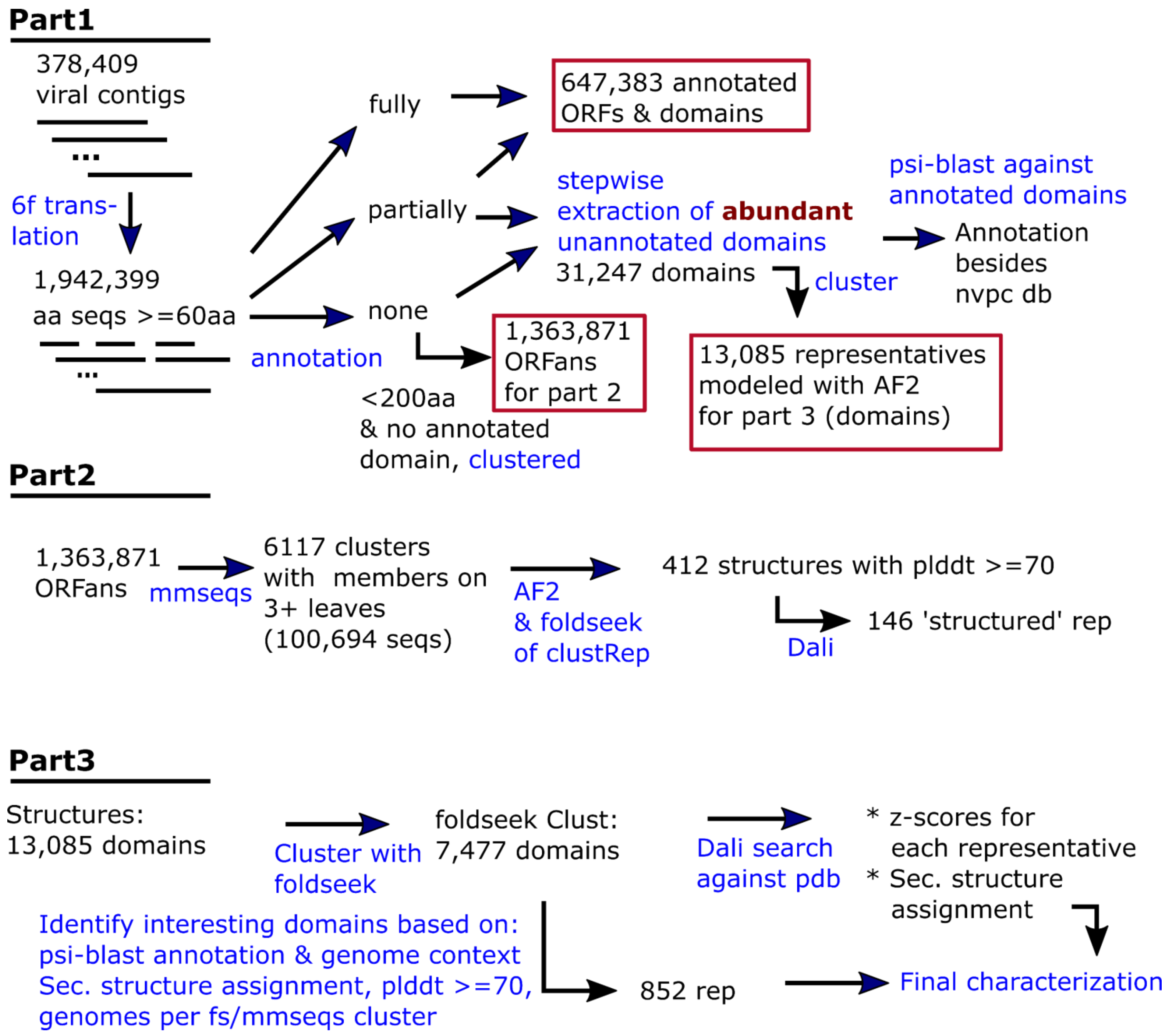

Fig. S2: ORFan and CUD clustering

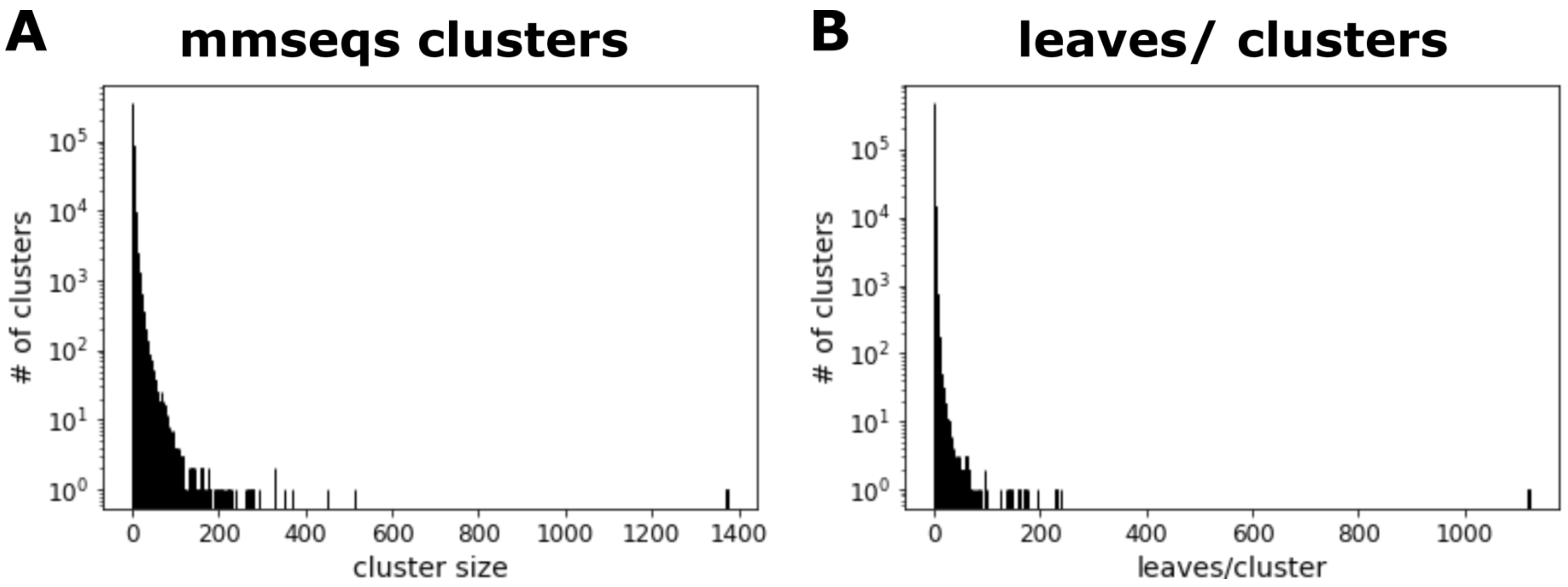

Fig.3S: Profile annotation coverage across ICTV exemplar protein clusters

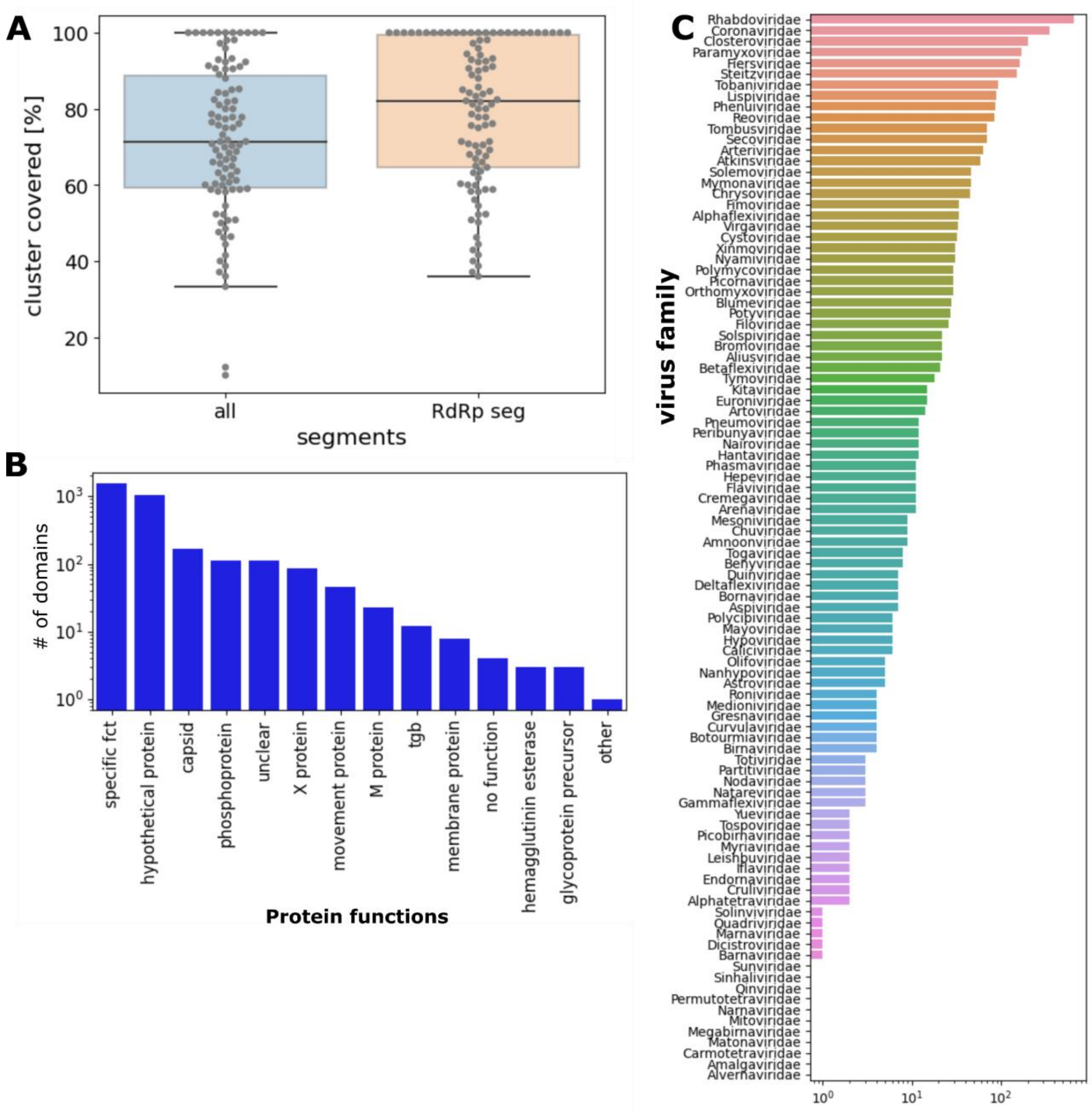

Fig. S4: Characteristics of ORFan structures

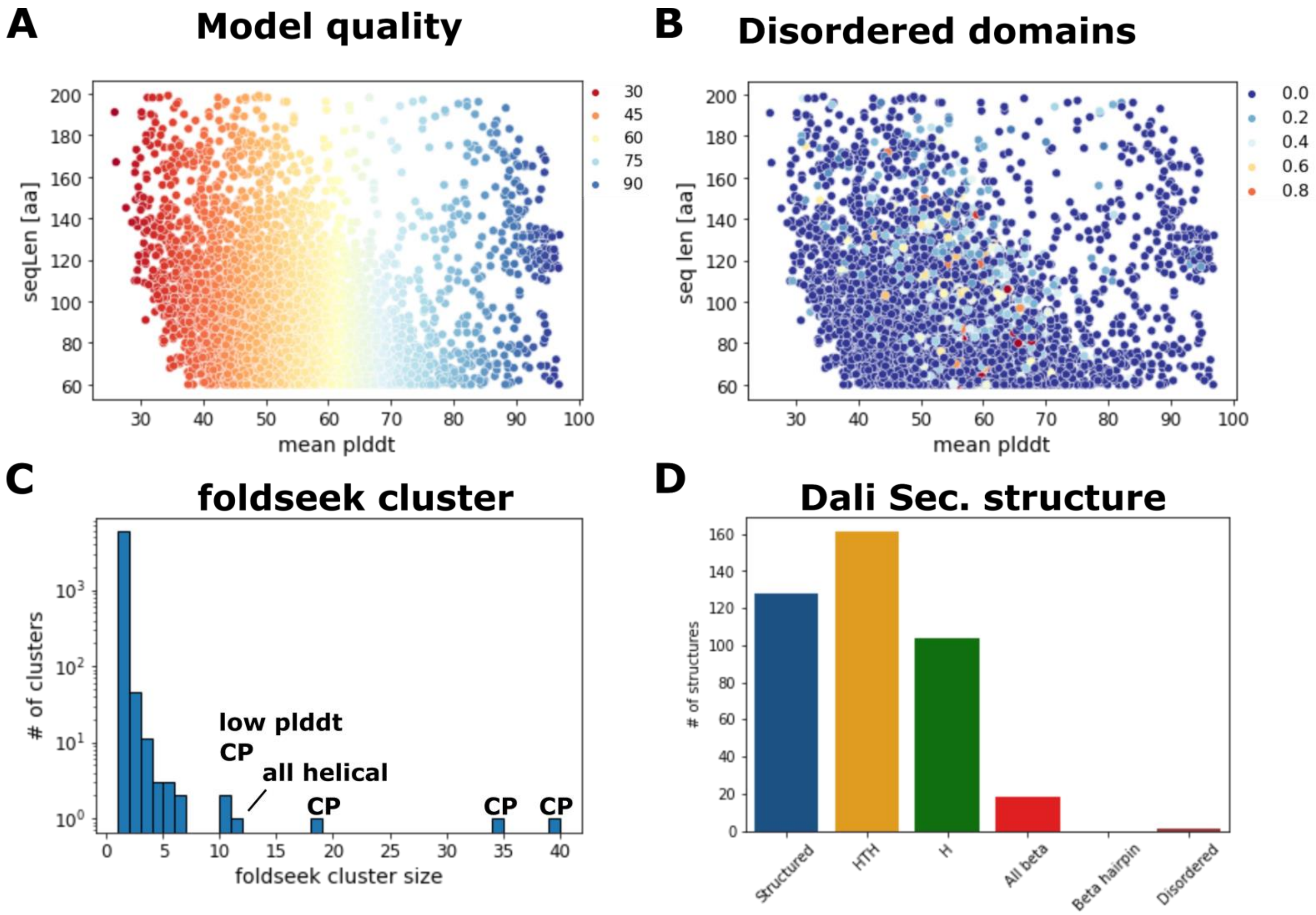

Fig. S5: Characteristics of un-annotated domain structures

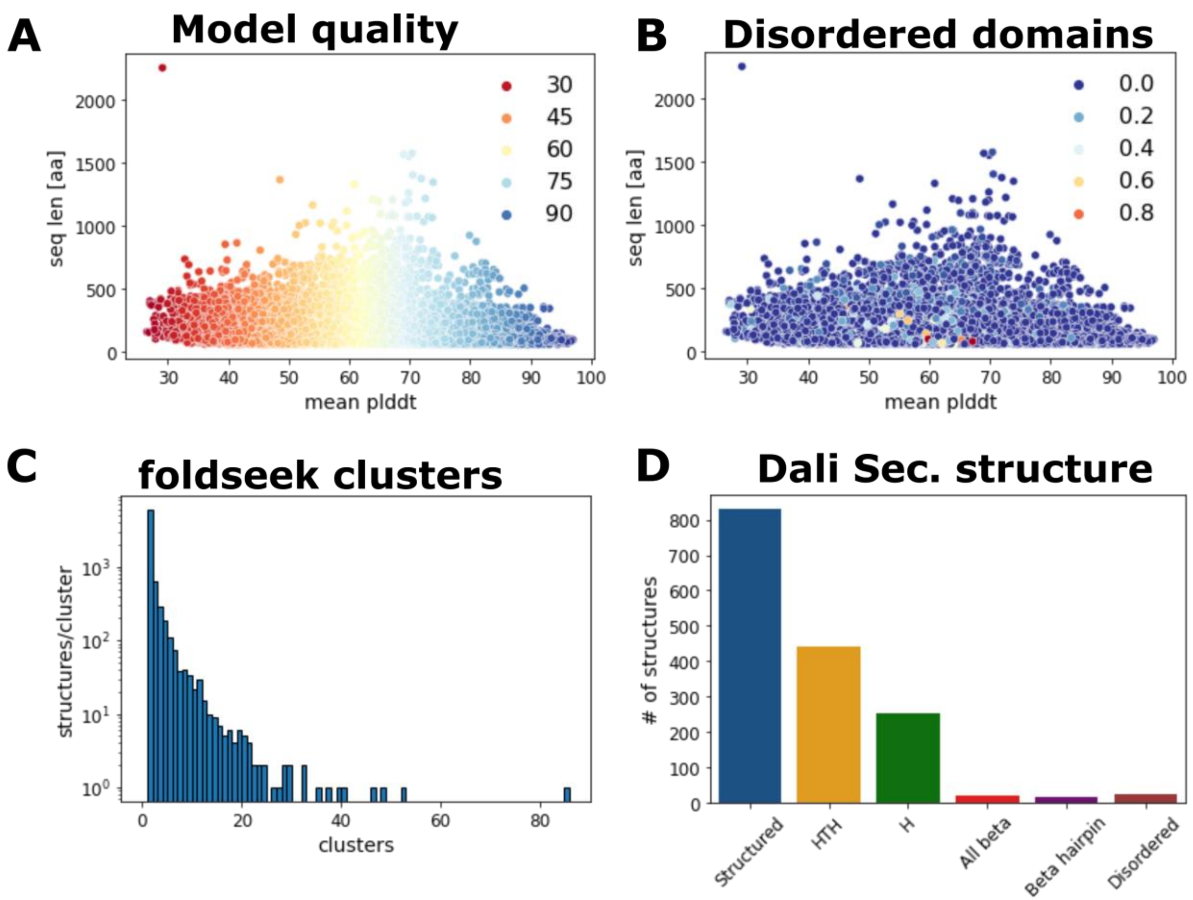

Fig. S6: Phylogenetic distribution of OOI and COI structure clusters

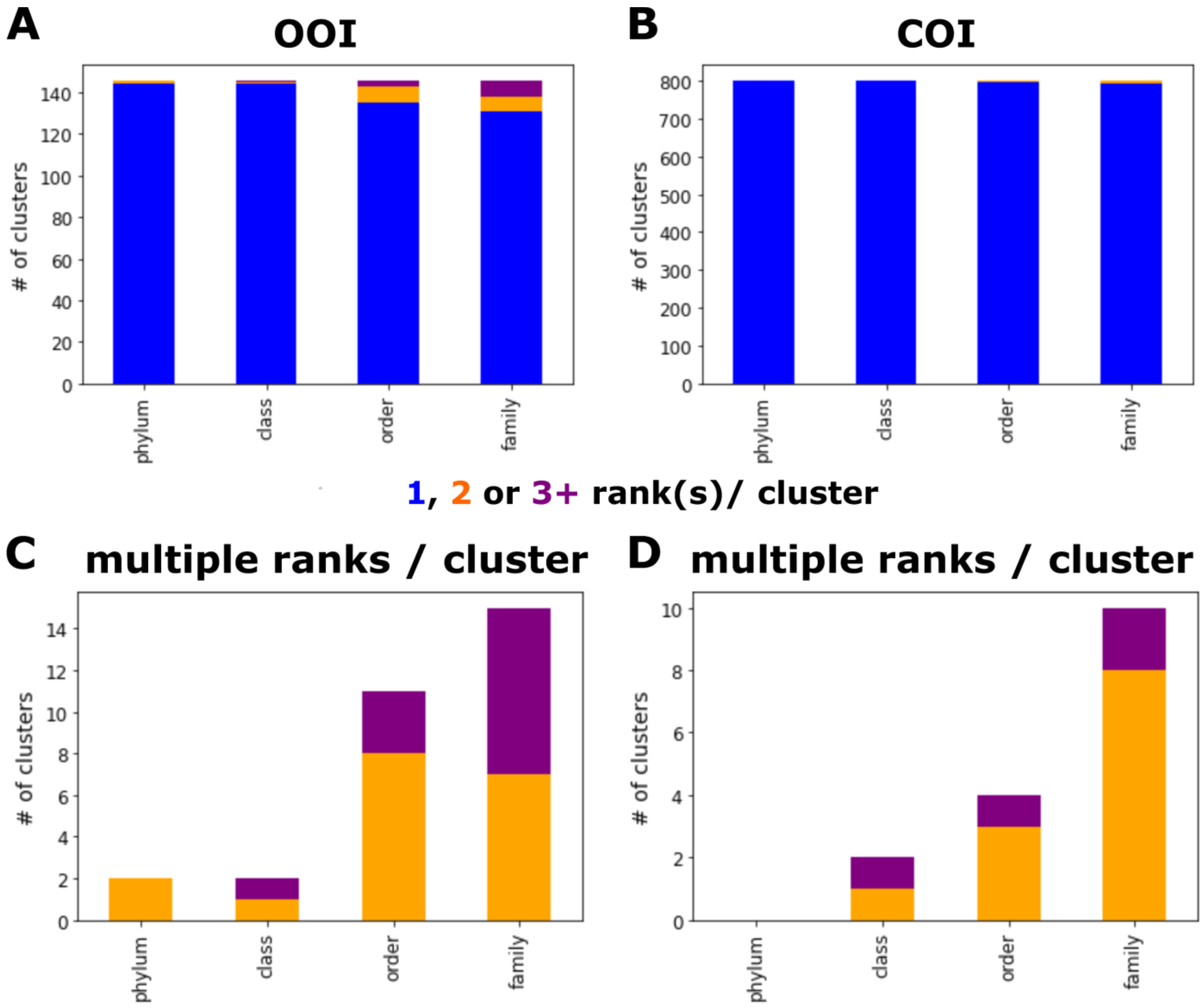

Fig. S7: Structurome clustering

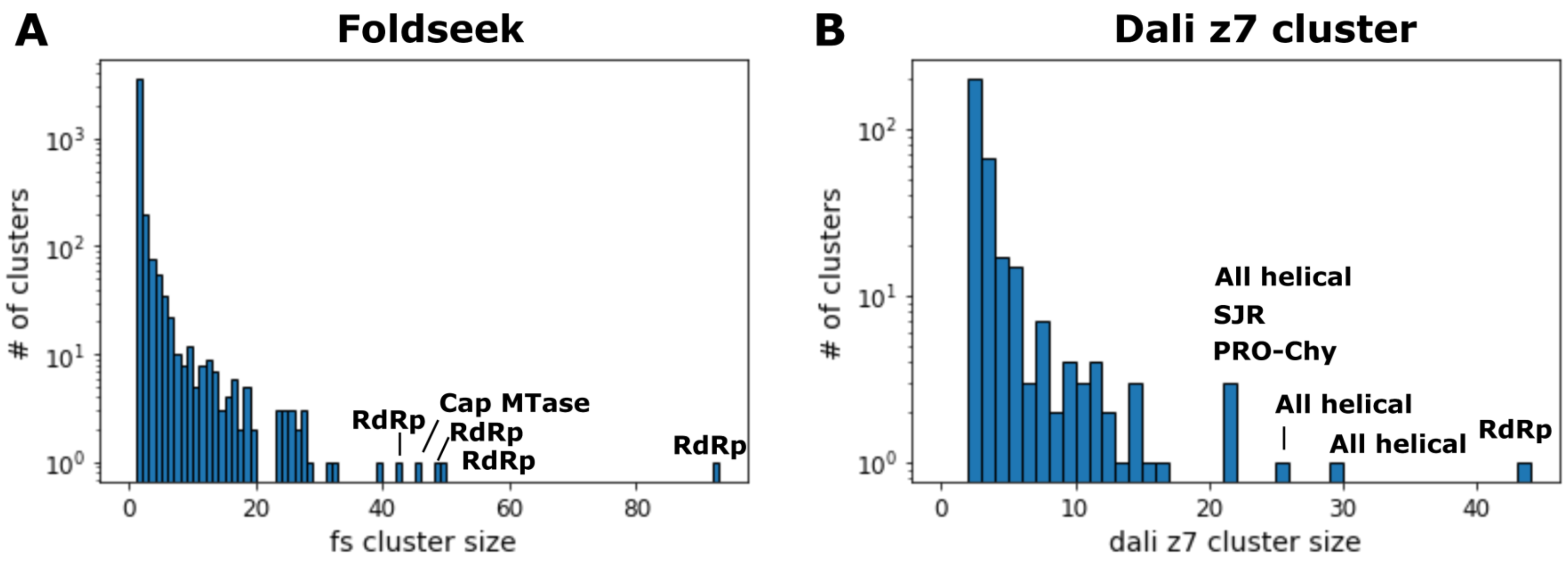

Fig. S8: Structurome as structure-structure network

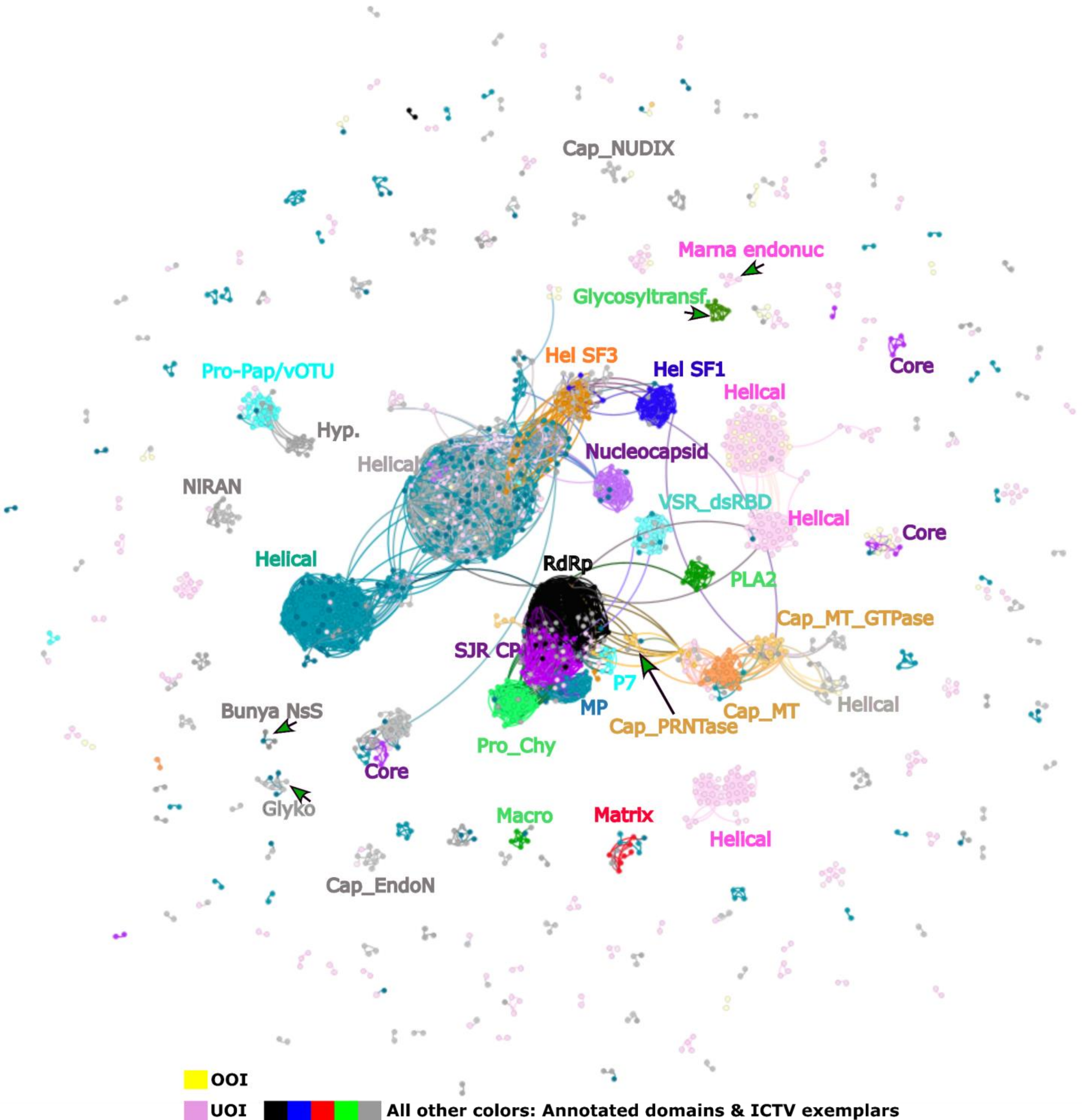

Fig. S9: Beta-barrel folds found in *f.0145*

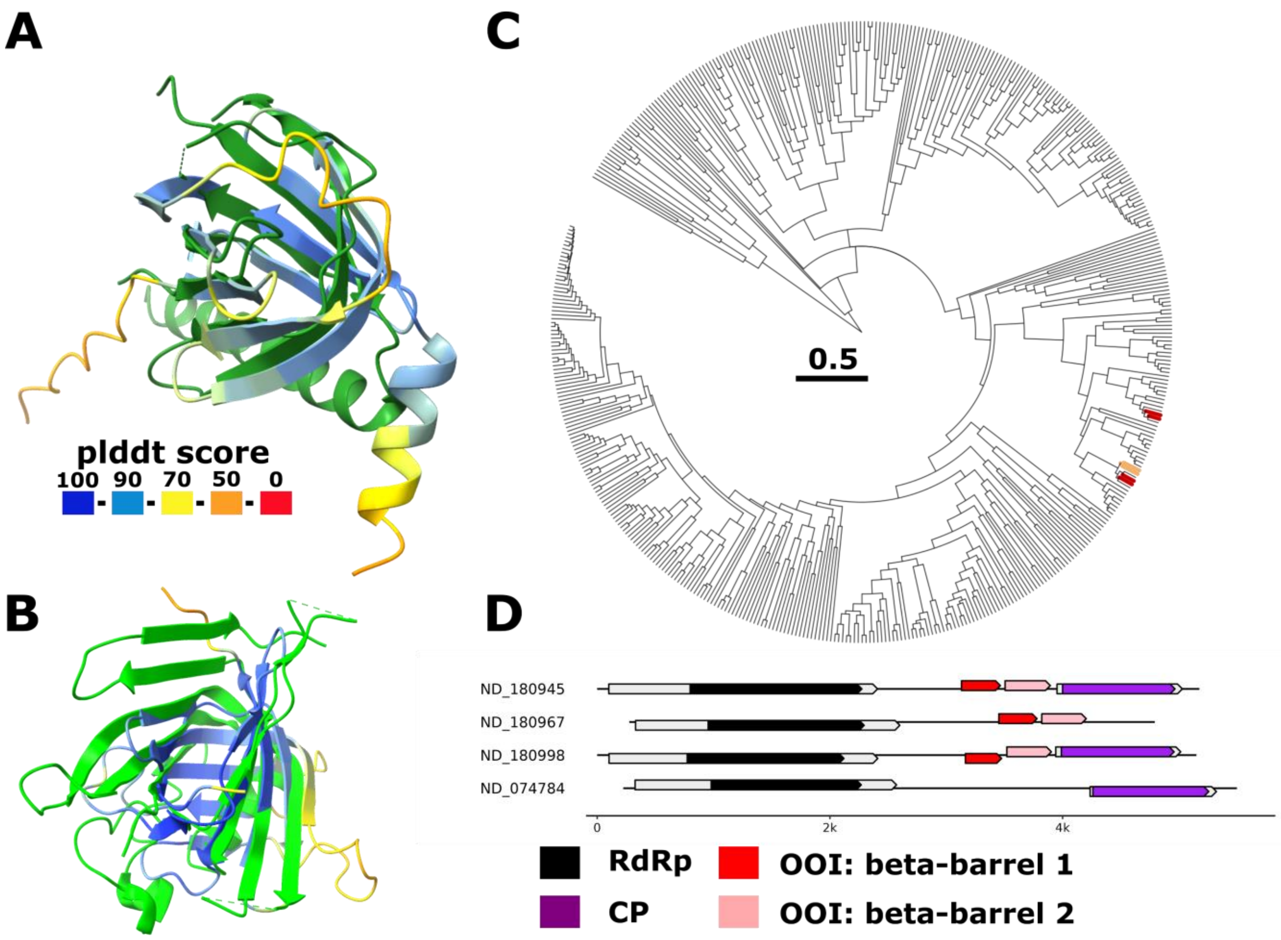

Fig. S10: Structure-guided alignment of viral core-p7 RBD-like folds and related cellular kinases

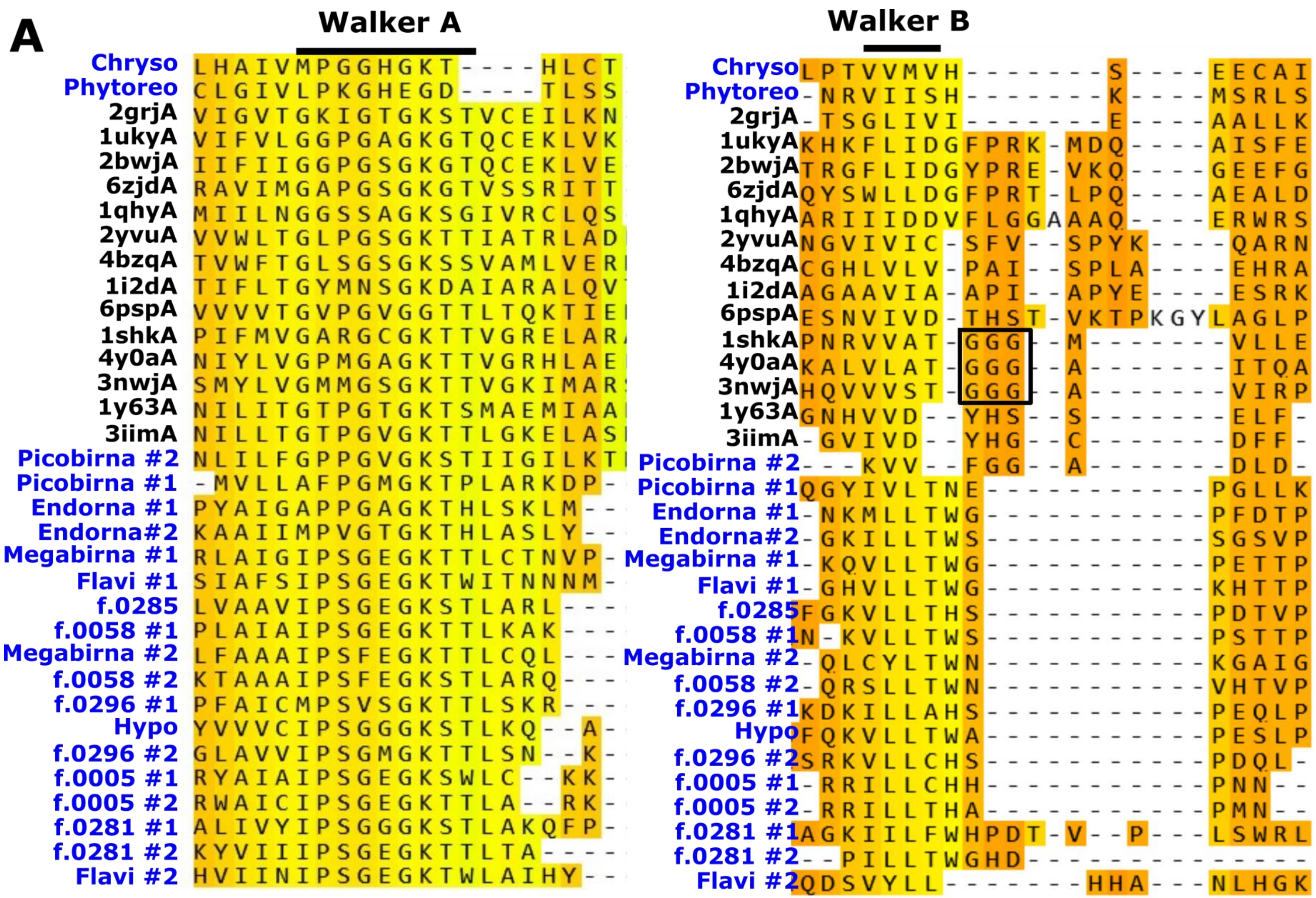

Fig. S11: Viral core-p7 RBD-like folds from various viral families and genome organisation

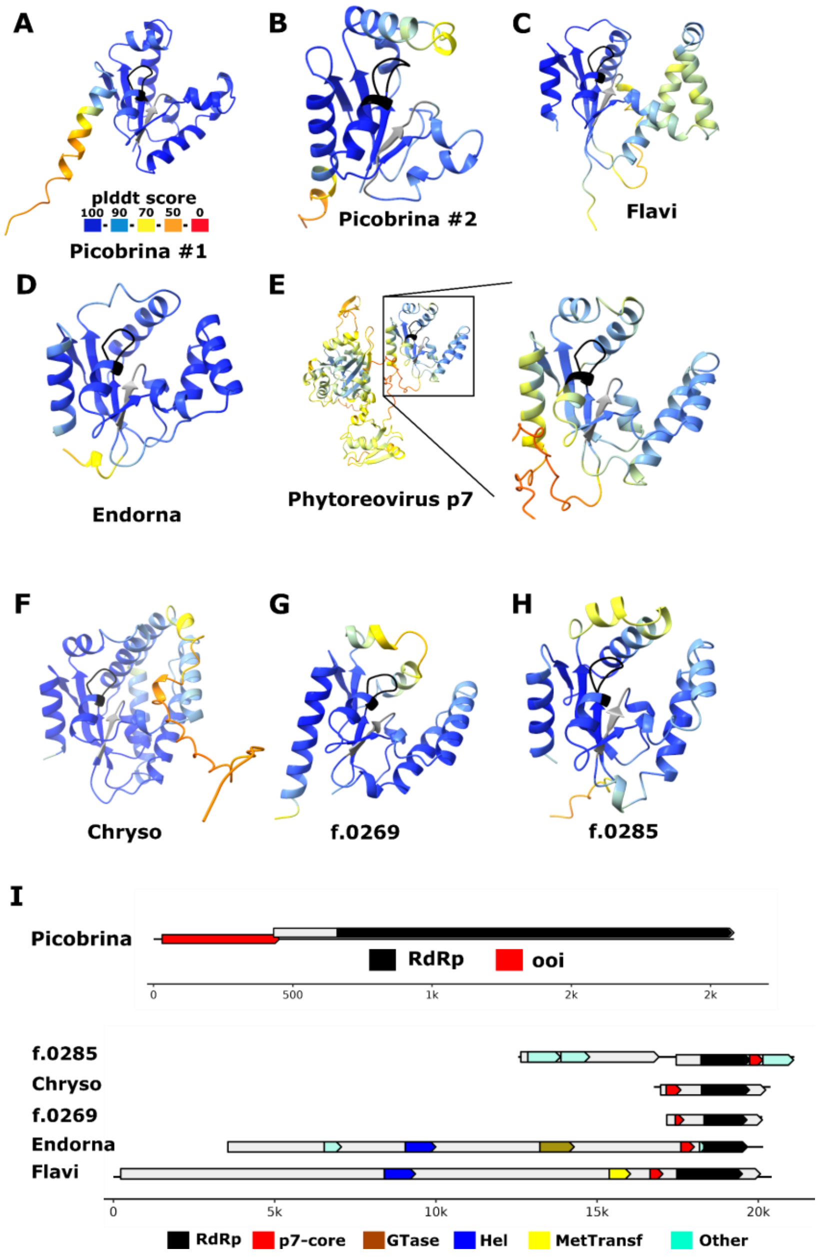

Fig. S12: VSR-like RNA binding domain in f.0092.base-Permutotetra

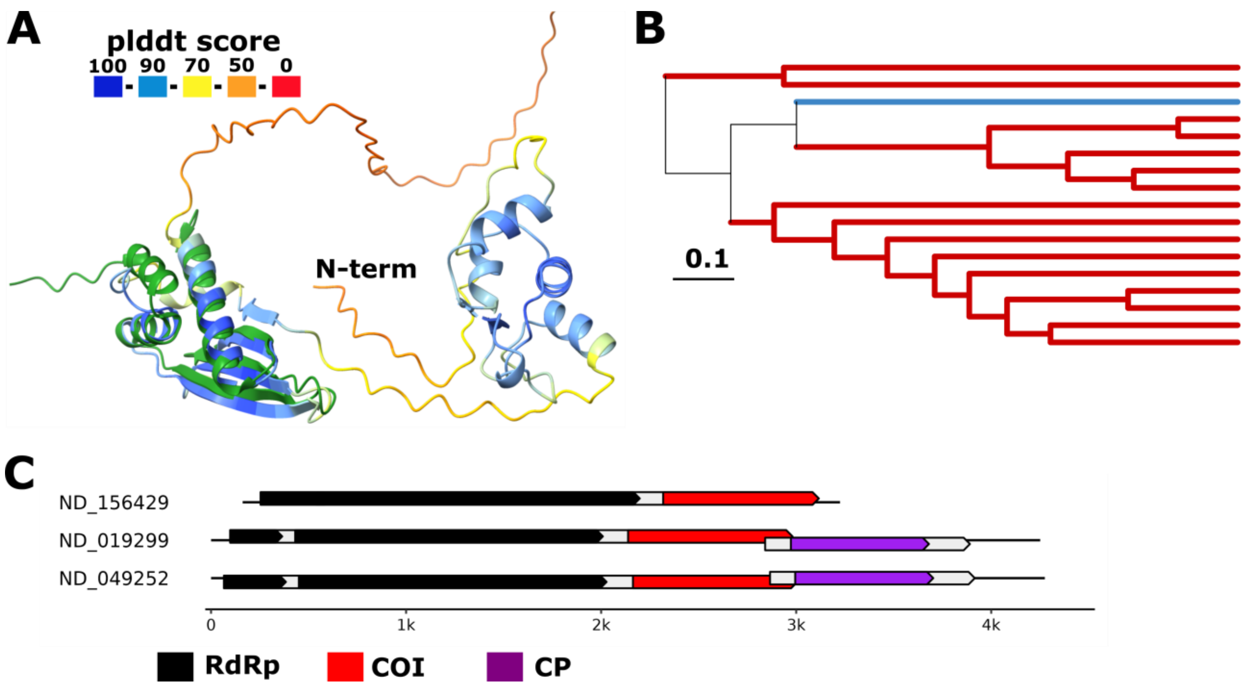

Fig. S13: Galactose binding domain in f.0008.base-Polycipi

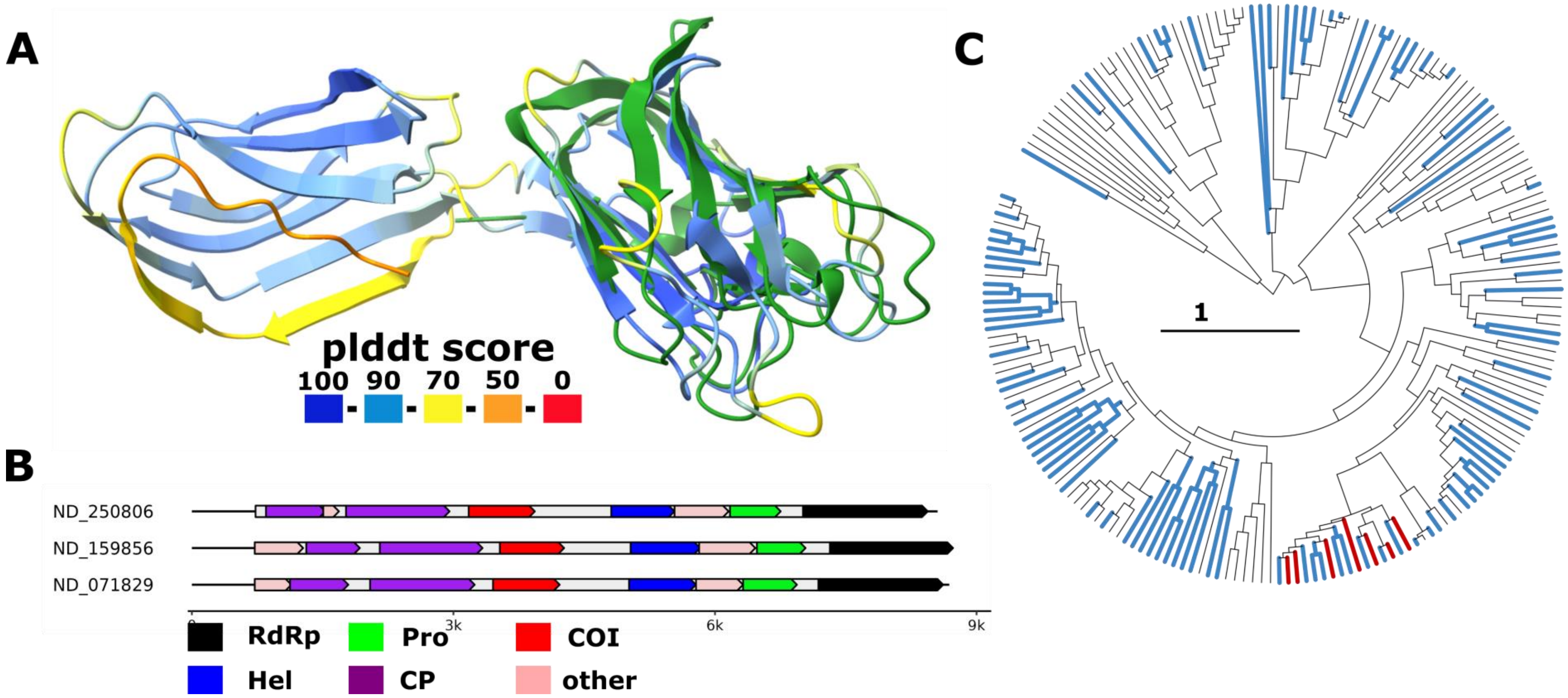

Fig. S14: Exonuclease & methyltransferase in f.0181

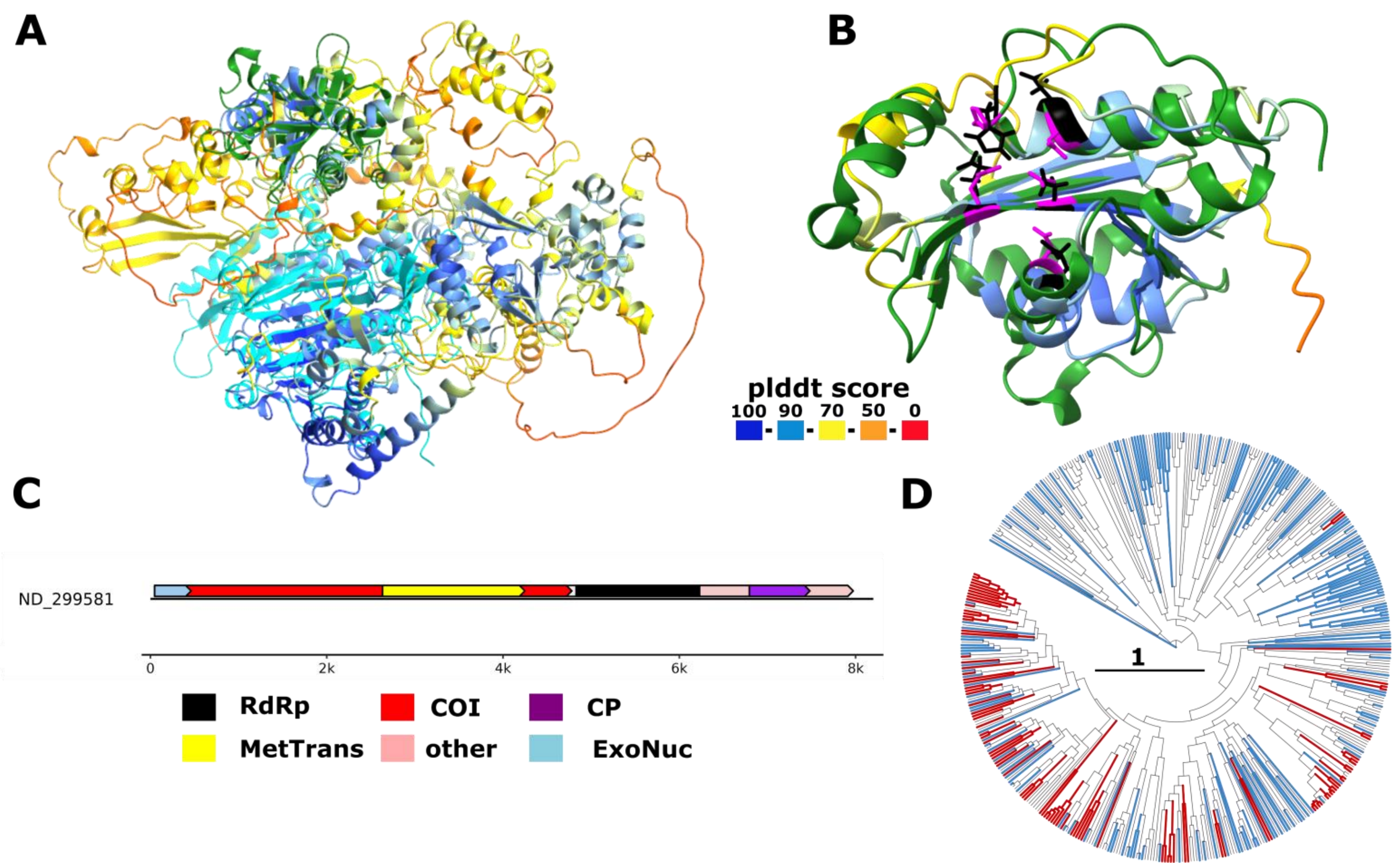

Fig. S15: Secovirus C-terminal domain of unknown function

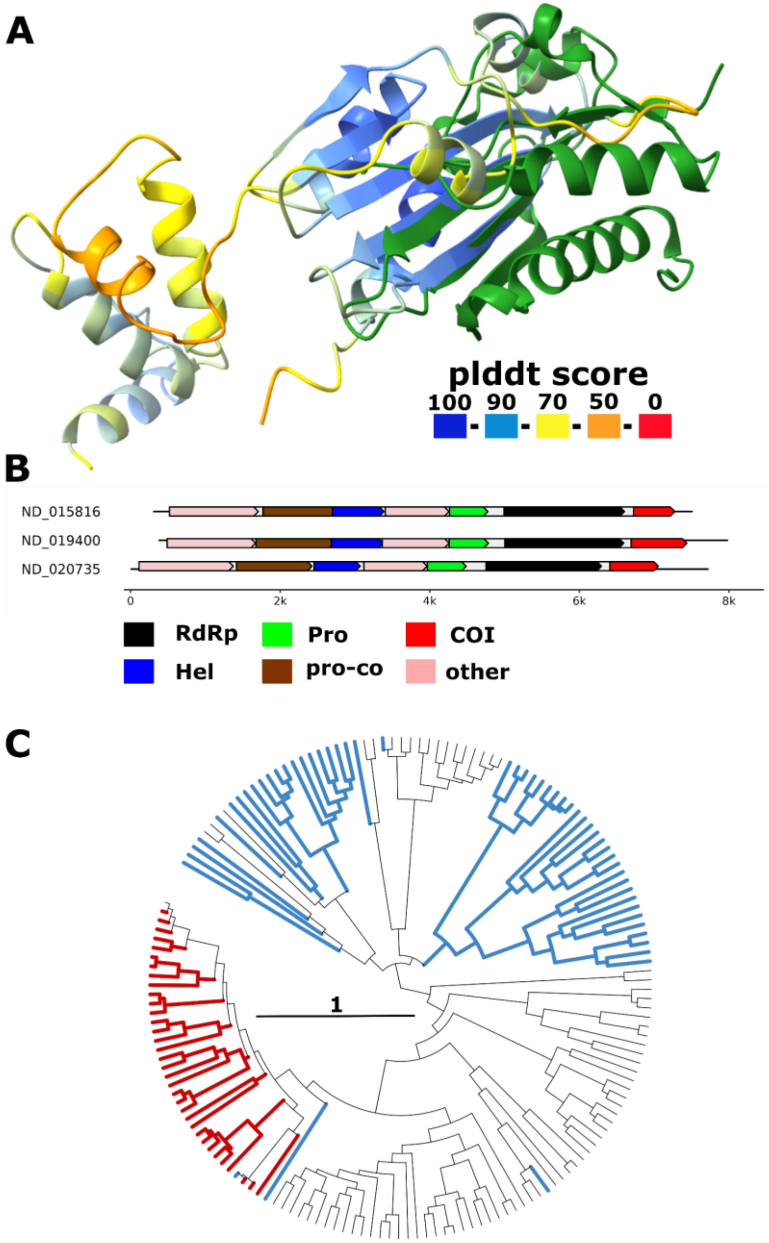

Fig. S16: alpha-beta fold OOI found in *Rhabdoviridae*

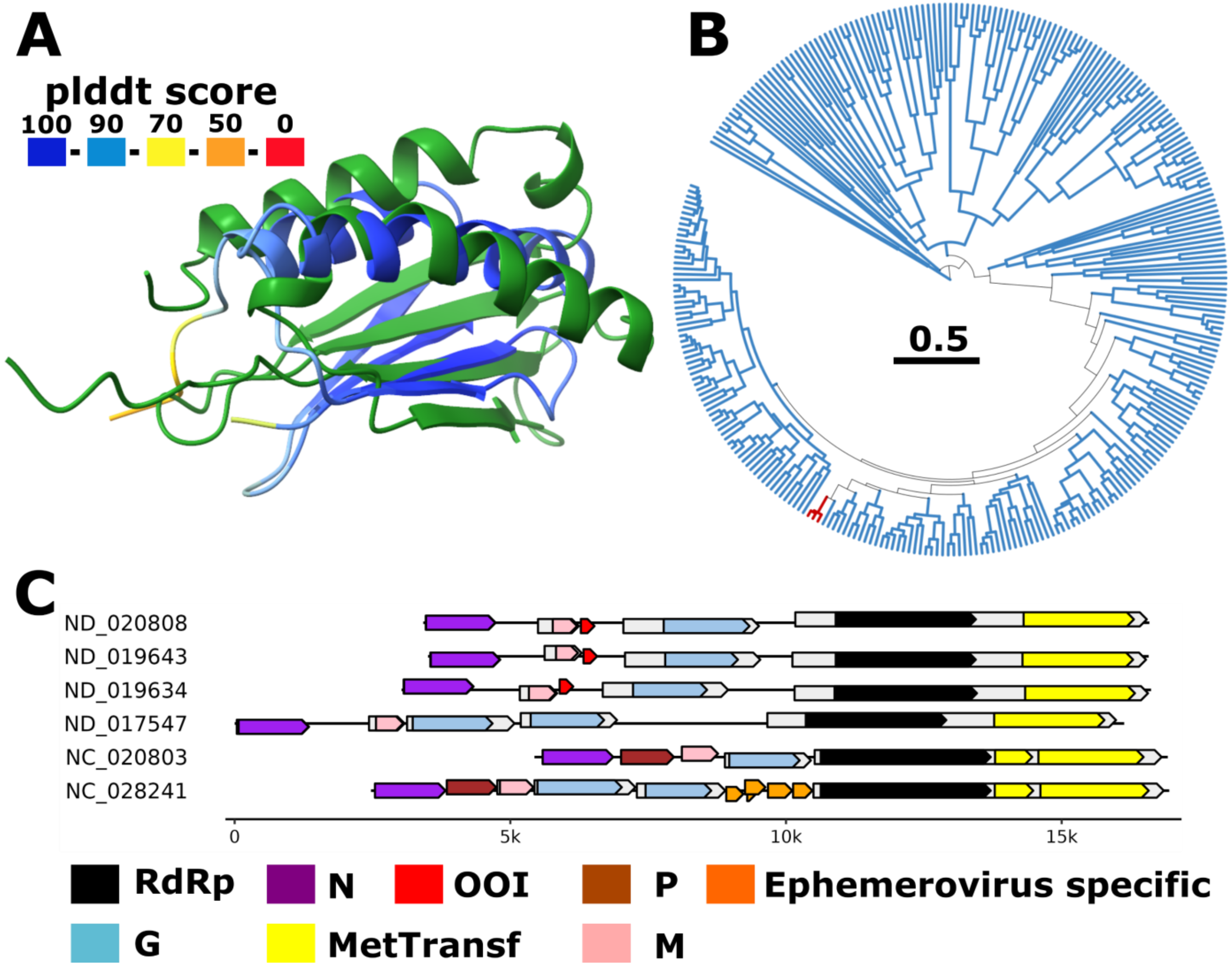

Fig. S17: vOTU in Deltaflexiviridae and related families

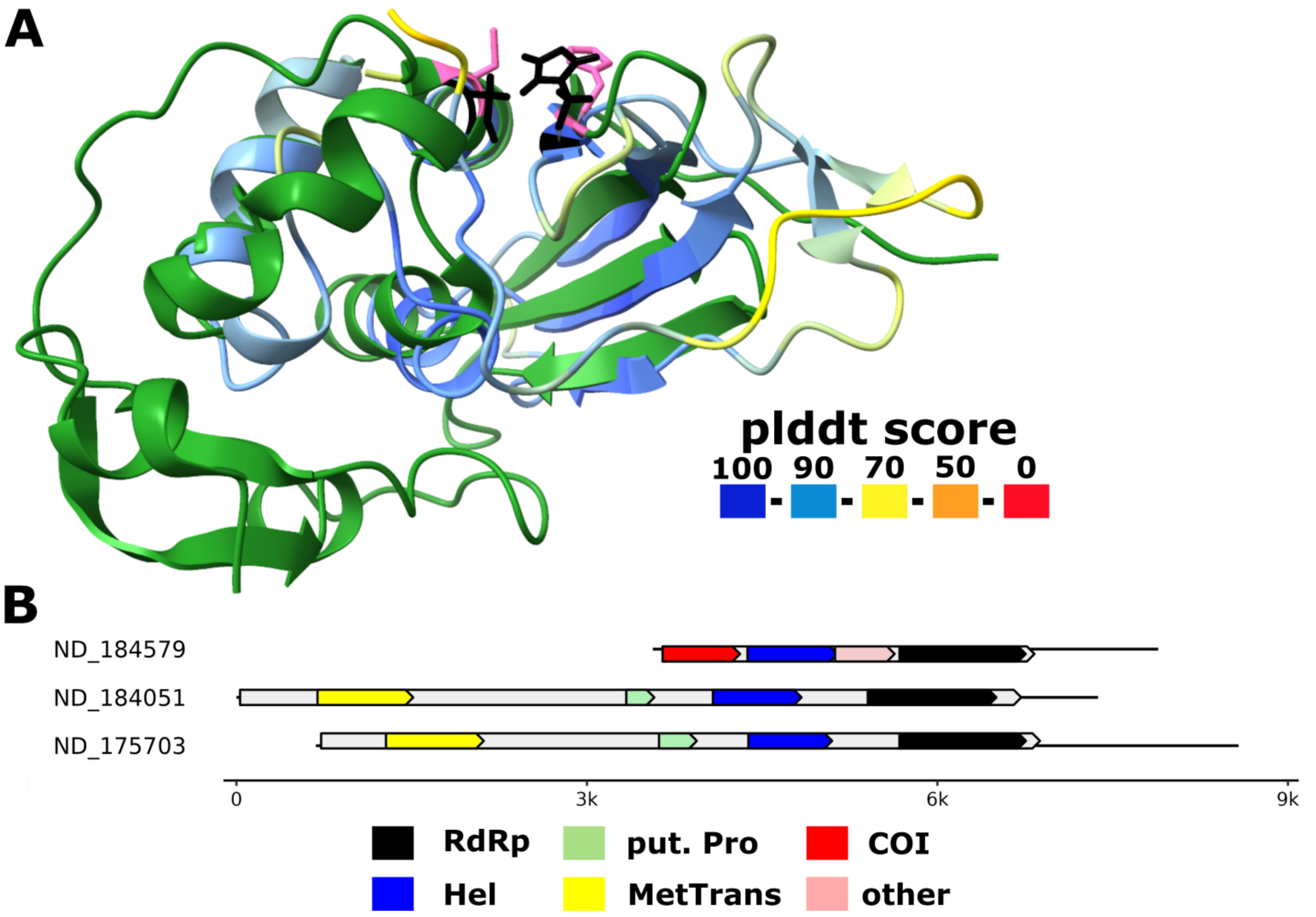

Fig. S18: Capsid protein in *f.0198*

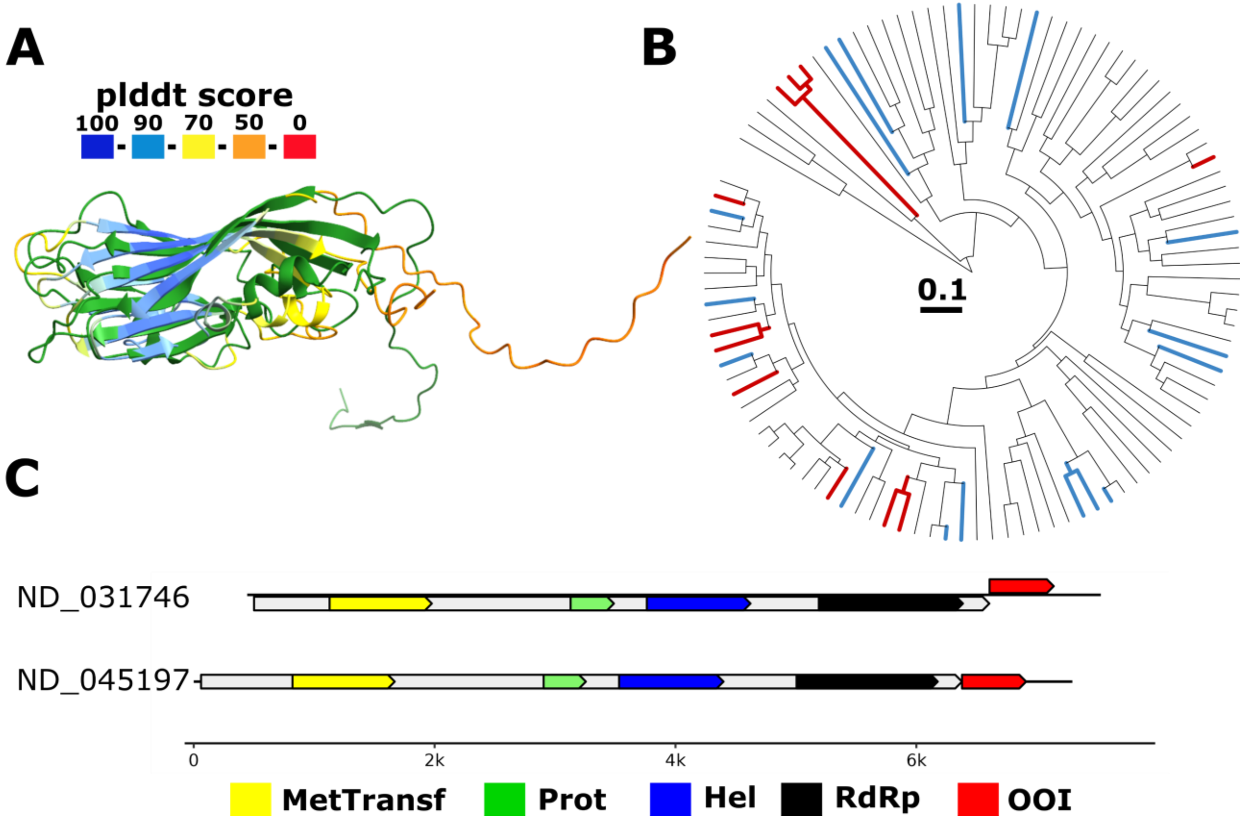

Fig. S19: Distinct domains per virus family

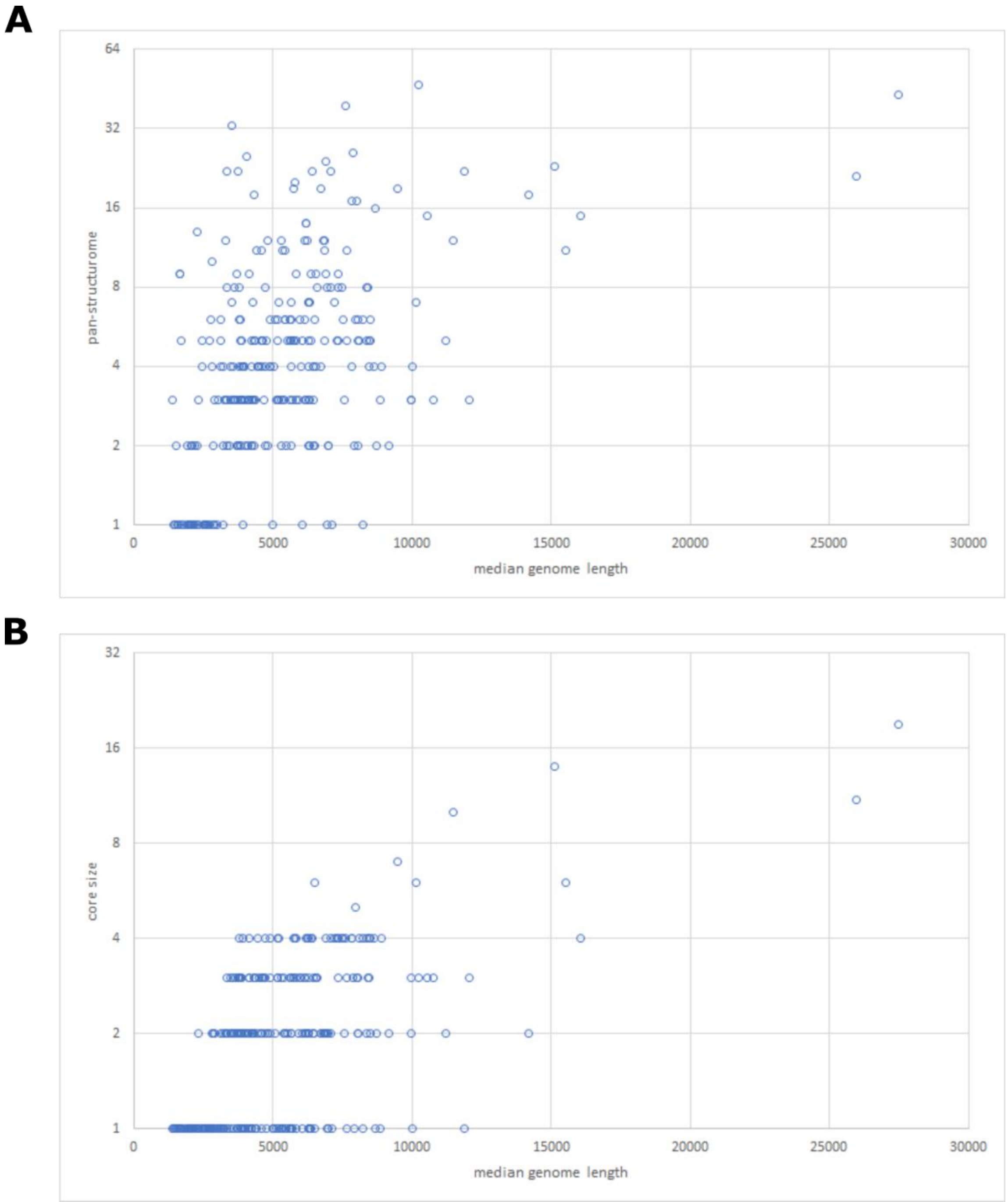

Supplement: Supplemental figures — Figures S1 to S19. [file mbio.03200-24-s0001.pdf]
